# Human papillomavirus vaccine delivery in Mozambique: identification of implementation performance drivers using the Consolidated Framework for Implementation Research (CFIR)

**DOI:** 10.1186/s13012-018-0846-2

**Published:** 2018-12-13

**Authors:** Caroline Soi, Sarah Gimbel, Baltazar Chilundo, Vasco Muchanga, Luisa Matsinhe, Kenneth Sherr

**Affiliations:** 1 Department of Global Health, University of Washington, Harris Hydraulics Laboratory, 1510 San Juan Road, Seattle, WA 98195 USA; 2 Health Alliance International, 1107 NE 45th St #350, Seattle, WA 98105 USA; 3 Universidade Eduardo Mondlane, Av. Salvador Allende no. 702, Maputo, Mozambique; 4 Health Alliance International, Rua Caetano Viegas no. 67, Maputo, Mozambique; 5 Department of Family and Child Nursing, University of Washington, Magnuson Health Sciences Building, 1959 NE Pacific St, Seattle, WA 98195 USA

**Keywords:** HPV vaccine, CFIR, LMIC, Mozambique, Gavi, Demonstration project

## Abstract

**Background:**

Since 2012 Gavi, the Vaccine Alliance has provided financial support for HPV vaccine introduction in low- and middle-income countries (LMICs); however, funding has been contingent on establishing a demonstration project prior to national scale-up, in order to gauge effectiveness of delivery models. Although by 2016, most beneficiary countries had completed demonstration projects, few have scaled up delivery nationwide. An important barrier was the dearth of published, country-specific implementation recommendations. We employed the Consolidated Framework for Implementation Research (CFIR) as a lens to identify drivers of heterogeneous (dissimilar) implementation performance during Mozambique’s 2-year demonstration project. Mozambique presents a compelling example as the country conducted demonstration projects in three different districts with extremely different economic resources and sociocultural practices.

**Methods:**

A post implementation interpretive evaluation was undertaken. Forty key informant interviews were conducted with district and health facility immunization staff, Ministry of Education managers, and teachers across the three demonstration districts, central level informants from MOH, research institutes, and immunization program partners. We compared valence and strength ratings of CFIR constructs, across diverse implementation sites, so as to explain drivers and barriers to implementation success. Two researchers coded separately, and subsequent content analysis followed pre-defined CFIR construct themes.

**Results:**

Eighteen constructs emerged from informants’ responses as implementation influencers. Adaptability was identified as an important construct because delivery modalities needed to meet differing levels of girls’ school attendance. Expanding outside of school-based delivery was needed in the low-performing district, making the vaccine delivery process more complex. Available resources varied across the three sites, with one site receiving direct Gavi support, while others received primarily state-based support. These latter sites reported considerably more implementation bottlenecks, in part related to weaker infrastructural characteristics and insufficient organizational incentives. Health workers’ beliefs in importance of vaccines and an organizational culture of making personal sacrifice for immunization program activities drove implementation performance. Advocacy and social mobilization through the right opinion leaders and champions generated higher demand.

**Conclusion:**

HPV vaccination presents a pertinent opportunity for the prevention of cervical cancer in Mozambique, sub-Saharan Africa, and other LMICs. However, important barriers to broad-scale implementation exist. We recommend the development of local and global strategies to overcome barriers and facilitate its expanded utilization.

**Electronic supplementary material:**

The online version of this article (10.1186/s13012-018-0846-2) contains supplementary material, which is available to authorized users.

## Background

Annually, half a million women worldwide are diagnosed with cervical cancer (CC) and 50% among them die from the disease [1]. The burden is higher in low- and middle-income countries (LMICs) where 85% of diagnosed cases and 87% of deaths occur due to late detection and limited treatment options [2]. The highest CC incidence and mortality rates are observed in sub-Saharan Africa where screening programs lag behind those in other regions [3]. Mozambique is placed second on CC burden country rankings with high age-standardized rates of 65.0 and 49.2 per 100,000 for incidence and mortality, respectively [4]. Research evidence from the country has demonstrated a relationship between HPV infection and cancer lesions consistent with global patterns which show that more than 70% of CCs are attributable to HPV types 16 and 18 [5]. One study among 262 women aged 14–61 years found that 40% had HPV DNA, 19% abnormal cytology, and 12% cervical neoplasia [6], while another study confirmed HPV 16 and 18 infection in cancer biopsies of 78% of cervical cancer cases that were evaluated [7]. CC prevention programs were traditionally composed of screening and early diagnosis only, [8] but now, the HPV vaccine has become an integral component of cervical cancer prevention programs in high-income countries [9]. The World Health Organization (WHO) recommends the adoption of HPV vaccine by LMICs where CC prevention is a public health priority [10]. In support of this recommendation Gavi, the Vaccine Alliance (Gavi), in 2011, made the decision to provide funding for eligible LMICs to introduce HPV vaccine into their national immunization program (NIP) schedules. Countries were however initially required to conduct demonstration projects prior to national scale-up, to evaluate and prioritize possible HPV vaccine delivery models [11]. The rationale for piloting was the scarcity of established health service delivery mechanisms for the novel target group of girls aged 9–14 years in these countries. Traditional routine national immunization programs’ vaccines target age group is that of 9–22 months [11]. Demonstration projects rolled out quickly with 23 countries completing pilots. Unfortunately, national scale-up failed to progress at the same pace and by December 2017 only six countries (Rwanda, Uganda, Bolivia, Guyana, Honduras, and Sri-Lanka) had transitioned from the pilot to national rollout. These fall far short from Gavi’s target of eight national HPV vaccination programs by December 2015 [12]. Mozambique is one of the countries that have been in a long transition phase. Demonstration projects were completed in three districts in November 2015, with a plan of commencing national scale-up in January 2018, but by December 2017, there had been no progress in the development of a national rollout strategy. There has been a lack of information about implementation from the three demonstration sites that allows understanding of potential barriers to implementation.

Our study aims to fill this identified information gap by providing salient implementation findings from Mozambique’s HPV vaccine pilots, to inform and, hopefully, expedite, national scale-up. In addition, Mozambique provides a unique example because while most other countries implemented only Gavi-funded pilots, the country included two government-funded sites.

Implementation research is concerned with the uptake of health interventions in different settings and the identification of context-specific implementation success barriers and facilitators. Key implementation determinants documented in the literature include acceptability, feasibility, appropriateness, costs, and fidelity [13–15]. With the aim of standardizing terminologies and approaches for identifying and documenting implementation determinants, the Consolidated Framework for Implementation Research (CFIR) was conceived through aggregation of content from a review of more than 500 implementation science (IS) theoretical frameworks literature sources. The CFIR is organized into five domains (*intervention characteristics*, *outer setting*, *inner setting*, *characteristics of individuals* and *process*) that are subdivided into 39 constructs [16]. Due to its generic nature, wide applicability, the provision of a support platform by its developers, and continued revision and updates, the CFIR has been proven appealing to IS researchers and its utilization has significantly expanded since it was first published in 2009 [17]. CFIR has been utilized to identify barriers and facilitators of health program implementation [18–20] and also solutions to barriers [21] in disparate settings. A recent systematic review documented more than 25 empirical applications of the framework. The researchers found that the CFIR has mainly been applied to guide study planning, data collection, and analyses; however, post implementation assessments could benefit from more CFIR research [17]. Additionally, to date, to the knowledge of the authors of this paper, the CFIR has not been used to study implementation of vaccination programs in LMICs, despite several studies employing it to explore HPV vaccine delivery in the USA [22–24]. By utilizing the CFIR, as a guiding theoretical framework in our study, we are adding to the growing body of knowledge of CFIR use in LMICs.

“The goal of this paper is to show the utility of CFIR in identifying and documenting implementation barriers and facilitators for the scale-up of interventions in LMIC health systems, such as that of Mozambique.”

## Methods

A post-implementation interpretive evaluation [25], which sought to explain implementation success or failure through the triangulation of project implementation stakeholders’ experiences, was carried out. The authors followed five steps as described below to apply the CFIR to the evaluation:

### Step 1: Defining the innovation

Prior to data collection, we defined the innovation of interest as the vaccine delivery model rather than the vaccine itself. The country chose to explore a periodic, school-based vaccine delivery model, on the premise that most of the target group, of girls aged 9–14 years, would be found in schools. Periods of 1 week were predetermined and utilized to deliver one dose of the vaccine, in an outreach format, to schools. Additionally, some community outreach visits were made in the days following the school-based vaccination. During the first year of the project, each eligible girl received three doses of the bivalent Cervarix™ vaccine, administered in May, June, and November to fulfill the WHO recommended schedule of 0, 1, and 6 months. In the second year, following a revision of WHO guidelines, doses were reduced to two, to be given at an interval of 0 and 6 months. These were administered in June and November to a new group of eligible girls. For outreach visits, teams composed of a health worker and an auxiliary staff member, from all health facilities in each district, visited up to three schools and two community locations, in the course of the five weekdays of the set vaccination week. In each school, one identified responsible teacher was tasked with registration of girls prior to the vaccination day. Additionally, on vaccination day, the responsible teacher arranged a vaccination venue and organized girls in queues for vaccine administration.

### Step 2: Defining the unit of analysis, site inclusion, and performance criteria

We defined the district as the unit of analysis for our study. There were only three district pilot sites and we decided to collect data from all of them, given that each district had been carefully selected for the demonstration project, to ensure representation of distinct sociocultural and economic realities in Mozambique. Health and socioeconomic parameters were only available at the provincial level and each demonstration project district represented one of the provinces shown in Table 1.

**Table 1:** Provincial health and socioeconomic parameters in the HPV demonstration project provinces

Parameters	Maputo	Manica	Cabo Delgado
Proportion of girls aged 6 years or more who enrolled in primary schools	64.7	63.5	46.8
Under-five mortality	96	114	116
Contraceptive prevalence rate among women 15–49 years old (married or in union)	32.8	12.5	2.9
Proportion of households with access to potable water	85.1	84.1	37.1
Proportion of households with access to electricity	60.3	22.2	5.0
Wealth quintile (proportion in poorest quintile)	1.2	5.5	23.8

Source: INE et al. 2013 (DHS 2011) [35]

**Fig. 1:**
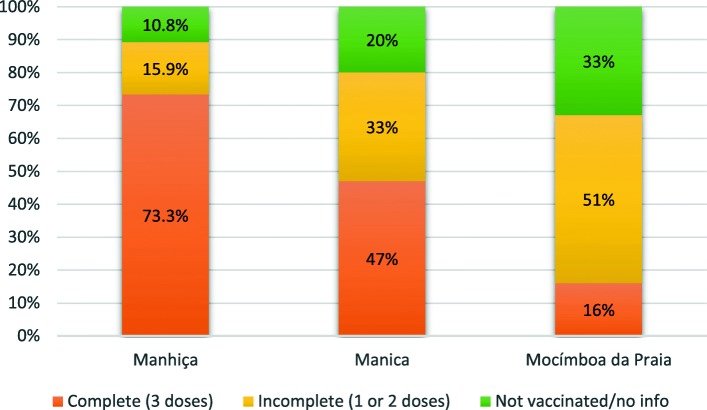
Mozambique demonstration project HPV vaccination coverage. Source, National Immunization Program 2015

We classified the sites as high, intermediate, or low performing based on HPV vaccination coverage, achieved during demonstration project implementation (Fig. 1).

### Step 3: Initial CFIR construct selection and data collection

In order to select study constructs, we mapped each CFIR construct to the Mozambique demonstration project setting. To achieve this, each CFIR description for all five domains (*intervention characteristics*, *outer setting*, *inner setting*, *characteristics of individuals*, and *process*) and 39 constructs were read through. Thirteen constructs did not match Mozambique’s setting and were deemed irrelevant and excluded at this point. Five of these were from the domain of *characteristics of the individual*, which we excluded because HPV vaccine delivery was being considered a systems level intervention and our primary interest was to provide guidance on how health systems could best deliver it to the target population. We then developed a semi structured interview guide to capture information on the selected CFIR constructs and used it to conduct 40 key informant interviews (KIIs) at the Ministry of Health (MOH) central level and all three demonstration districts. Saturation was prevented through purposive sampling of study participants, who were selected based on their institutional positions and roles during implementation of HPV vaccination. They included, teachers, health facility immunization staff, district immunization and education managers, provincial immunization staff and medical heads, and national immunization program staff, as well as staff from research institutions and non-governmental organizations (NGOs) who had supported the NIP throughout all demonstration project implementation phases. The interviews were conducted by four of the authors in person at the interviewee’s venue of choice. Interview sessions lasted between 1 and 2 h and voice digital recordings were obtained and then transcribed verbatim.

### Step 4: Final CFIR construct selection

The final constructs for analysis were selected through the coding process. The CFIR codebook template, which provides codebook descriptions for each construct, guided the development of our study codebook. Using Nvivo software version 11, responses to the research questions we had created for 26 CFIR constructs, deemed relevant in step 3, were sought and coded in the KII transcripts. A team of two researchers coded separately, and the kappa statistic was used to measure inter coder reliability at a threshold of 80% [26]. A report of each code was produced, read, and re-interpreted in order to create a synthesis of findings for each CFIR construct [27]. At this point, we eliminated constructs that were not reflected in our transcripts and remained with 19 constructs for the next step.

### Step 5: CFIR construct valence and strength rating

Ratings were performed to determine valence, which assesses whether the construct had a positive, neutral, or negative influence on implementation performance, and strength which is the degree of its influence. For this purpose, valence rating definitions, set on a scale of − 2 to + 2, were utilized together with strength ratings as described on cfir.com website [28]. Constructs for which respondents described as negatively influencing on performance with explicit examples received a − 2 score, while those that positively influenced performance and respondents provided explicit examples, received a + 2 score. For interpretation, a comparison of ratings across the units of analysis (demonstration districts) was conducted using an excel-based rating matrix. Finally, the pattern and strength of ratings for each implementation district were established. In so doing, we determined which constructs distinguished performance, either strongly or weakly, and identified constructs that did not distinguish but influenced performance either negatively or positively as well as those that were neutral, neither distinguishing nor influencing performance.

Trustworthiness of the data was ensured through triangulation of data collection methods (individual interviews, direct observations, and document review), through multiple coders and use of intercoder reliability (as described above). Validity was guaranteed by using appropriate data collection methods for each type of data and by obtaining stakeholder feedback on the data through the presentation of preliminary findings.

## Results

A total of 19 constructs were evaluated for valence and strength, 12 distinguished performance and were classified as distinguishing (11 strongly and 1 weakly). Five were not distinguishing but positively influenced performance, one not distinguishing but negatively influencing, and one was neutral neither distinguishing nor influencing implementation performance (Table 2).

**Table 2:** Mozambique HPV vaccine demonstration project CFIR construct valence rating

Evaluated Constructs		Valence		
	Distinguishing	High	Intermediate	Low
Innovation characteristics				
Adaptability	Strongly	+ 2	− 2	− 2
Complexity	Strongly	− 1	− 2	− 2
Design quality and packaging	Not	+ 2	+ 2	+ 2
Outer setting				
Patient needs and resources	Strongly	+ 2	− 2	− 2
Inner setting				
Structural characteristics	Strongly	+ 2	− 2	− 2
Networks and communications	Weakly	− 1	− 2	− 2
Culture	Not	+ 2	+ 2	+ 2
Relative priority	Not	+ 2	+ 2	+ 2
Org. incentives and rewards	Strongly	0	− 2	− 2
Learning climate	Strongly	+ 2	0	0
Available resources	Strongly	0	− 2	− 2
Access to knowledge and info	Strongly	+ 2	− 1	− 1
Process				
Planning	Not	− 1	− 2	− 2
Opinion leaders	Strongly	+ 2	− 2	− 2
Champions	Not	+ 2	+ 1	+ 1
Key stakeholders	Strongly	+ 2	0	0
Innovation participants	Strongly	+ 2	0	− 2
Executing	Neutral	0	0	0
Reflecting and evaluating	Not	+ 2	+ 2	+ 2

Details of the findings are described below, categorized by each construct that was rated for valence and strength. (CFIR construct definitions can be found in Additional file 1: Table S1).

### Innovation characteristics

#### Adaptability (strongly distinguishing construct)

The demonstration project, vaccine delivery model, was designed at central MOH level to be primarily school based. The design did not consider nor address existing differences in proportion of girls enrolled in schools in the disparate demonstration districts, each of which presented a different sociocultural and economic setting. Detailed guidance was provided to districts on procedures to be followed during administration of HPV vaccine in schools but the community outreach component was left ambiguous. There was no clarification on roles of community structures and no variation in number of days allocated for community outreach visits in the different districts. The model did not make a provision for districts to adapt to their local context’s school enrollment situation. Key informants from the intermediate- and low-performing districts expressed the need for a delivery model that could cater for such differences. They stated that districts with a significantly larger proportion of out-of-school girls may require a different community outreach strategy to reach them. For example, they may need more community outreach days which in turn would mean a larger budget.

Here in Cabo Delgado we have a significant proportion of girls that are not in schools. Early marriages are the norm, so we have to be able to offer the vaccine in the schools, community and health facility for a longer period than the time that was stipulated for us during the demonstration project. (Provincial Health Directorate)

#### Complexity (strongly distinguishing construct)

The non-health facility nature of the demonstration project’s HPV vaccine delivery model created a complex working environment for health workers from all demonstration districts. Difficulties narrated by study respondents included the necessity to closely collaborate with the Ministry of Education and other non-health sector structures, and demand generation for the novel target age group and the location of a significantly larger number of girls not enrolled in schools in the intermediate- and low-performing districts. These shall be delved into within the *needs and resources of those served by the organization* construct. Health workers had to rely on teachers for school-based and community leaders for community-based vaccination. These non-health system individuals were expected to assist with certain activities such as pre-vaccination registration of girls and identification of a vaccination venue; in some instances, teachers took on the role of filling in girls’ vaccination cards or (and) vaccination registration books. District respondents stated that central level NIP did not define roles for non-health workers and they had to innovate methods for collaborating with teachers and community leaders. Such methods however were not always successful and impacted on their ability to reach all girls eligible for vaccination.

Vaccinating in schools, it is not an easy job because the social mobilization we have to conduct is different from what we normally do for vaccination. In this case we had to depend on teachers to communicate to girls, register them and also to organize them on the day of the vaccination. I had to keep calling the teacher using my mobile phone all the time which is different from what we do for the usual vaccines we give to children here in the health facility (District Health Directorate)

Difficulties encountered with demand generation will be delved into in the *innovation participants* construct.

#### Design quality and packaging (non-distinguishing construct)

All demonstration districts received the same project materials from the NIP central level. Respondents from all demonstration project districts found the materials very useful in provision of vaccination procedures’ guidance. For this reason, this construct did not distinguish, but strongly influenced performance positively.

### Outer setting

#### Needs and resources of those served by the organization (strongly distinguishing construct)

This construct, which describes the extent to which vaccine recipients’ needs are accurately known and prioritized, as well as the barriers to reaching them, strongly distinguished performance. The low- and intermediate-performing districts’ health worker respondents reported a failure of the delivery model design to address some elements that subsequently compromised their ability to reach all eligible girls in their districts. Two main issues were raised, first, was the lack of knowledge of the exact location of girls who were not in school. While health workers were cognizant that these girls were in the community, not knowing their precise location became a barrier to reaching them. They said they would need to rely on community and neighborhood leaders to assist them in identifying where these girls could be found and similarly where to best locate community outreach vaccine delivery points.

Girls not in school, where are they? It will be best to collaborate with community leaders to have them identify the best locations to offer the vaccine so that we can reach most girls out of school. (District Health Directorate)

On the contrary health worker key informants from the high performing site said they did not have a problem locating the few girls who were not enrolled in schools in the community.

Another barrier to reaching the eligible target group was a vaccination date scheduled during a local public holiday. Local public holidays known as city commemoration days fall on different calendar days for cities in Mozambique. The HPV vaccination date that was selected at the central level was applied uniformly to all districts with no consideration of the occurrence of a localized city commemoration day. Key informants from the intermediate- and low-performing districts mentioned this as a difficulty that contributed to their sites’ sub optimal performance. The high-performing district health workers did not cite this problem.

### Inner setting

#### Structural characteristics (strongly distinguishing construct)

Some structural characteristics of the intermediate- and low-performing districts were stated by KIs as factors that negatively affect the ability of their site to achieve higher coverage of HPV vaccination. Specific examples were total number of schools in the districts transportation infrastructure such as state of roads and existence of bridges. Bridges are an important part of infrastructure in Mozambique because rivers and other water bodies are common features in most of the country. The lower socioeconomic development of these districts was an underlying factor determining the state of the transportation network. As shown in Table 1, the intermediate- and low-performing districts are economically less developed than the high-performing district. Interestingly, key informants from the high-performing district noted vaccination coverage variations with more economically developed areas attaining better performance than poorer areas.

even though the overall coverage here in the district was 70%, when we stratify, we find that areas that are economically advantaged had higher coverage rates (District Health Directorate)

The low-performing district had fewer schools resulting in a higher number of girls unenrolled in school, and schools were also of further distances from health facilities, resulting in increased travel time for school-based vaccination efforts. In addition, the low-performing district included islands, and thus, the ocean contributed to access challenges.

#### Networks and communications (weakly distinguishing construct)

The intermediate- and low-performing health directorate respondents cited non-transparent communication from central level MOH managers regarding financing. Specifically, information on when and how much demonstration project activities’ funds would be transferred to districts, were vague. The districts were repeatedly advised that funds would “arrive in a few days” but subsequently only arrived after the start of vaccination. However, health workers in the high-performing district did not experience this challenge because their funds came from Gavi and were, appropriately, disbursed prior to initiation of vaccination activities.

The central level kept telling us the funds will be in the province tomorrow, but we didn’t’ receive anything and finally we managed to get some funds for fuel but not per diems for the workers (Provincial Health Directorate)

#### Culture (non-distinguishing construct)

Organizational culture, which in this study refers to the culture and practices of the Mozambican MOH workers, was a non-distinguishing factor. Nevertheless, it strongly influenced implementation in a positive manner. Health workers at all levels (health facility, district, province, and central), of the Mozambican health system abide by a culture of resilience whereby making do under challenging circumstances is the norm, including sacrificing when a matter is deemed to be of national priority or interest. Health worker respondents from all sites stated that they went out of their way to ensure the demonstration project was implemented within planned timelines and they viewed their selection as pioneer HPV vaccination district as an honor.

We tried to explain to them (community leaders and teachers) that this was something that was going to benefit the population and our province had been selected for the demonstration project, that we just had to sacrifice by being a bit patient and doing what we can to make it work. (Provincial Health Directorate)

#### Relative priority (not a distinguishing construct)

This was not a distinguishing construct; however, it strongly influenced implementation positively. Health worker respondents, in all demonstration districts, perceived vaccination as a highly efficient disease prevention intervention, which should be prioritized.

#### Organizational incentives and rewards (strongly distinguishing construct)

HPV vaccination coverage of eligible girls in the government-funded districts, where incentives were not paid, was much lower than the coverage reported in the high performing district, where financial support from Gavi ensured that all individuals supporting the demonstration project implementation received incentives. The lack of timely financial support in the low-performing district resulted in negative repercussions for HPV implementation and acceptance. Disgruntled community leaders lost trust in the local health directorate who they believed had received money from the central MOH level to pay for community leader support, but were refusing to disburse it. Consequently, they not only failed to complete tasks they had agreed on (pre-vaccination registration of girls) but went further and used their important positions as opinion leaders, to discourage parents from allowing their daughters to be vaccinated, further diminishing implementation effectiveness.

The non-payment of some of those involved, led to discontent, dissatisfaction and even mistrust. Mistrust because they thought that we (the provincial health directorate) had received the money and had not paid them. Especially non-health people such as the teachers and community leaders who were helping us implement the program. (Provincial Health Directorate)

#### Learning climate (strongly distinguishing construct)

The learning climate is defined as the extent to which evaluation is integrated in demonstration project. The high-performing district (Gavi-funded) included an evaluation component while the intermediate and low-performing districts did not. This high-performing, Gavi-funded district subcontracted the local research institution (a demographic surveillance site based in the district) to carry out concurrent feasibility and coverage studies. A criteria to secure future Gavi funding for national HPV vaccination scaling included attainment of 50% coverage of eligible girls in the Gavi-supported district. Thus, realization of this goal became a country priority that consequently, strongly influenced performance in this site.

we (the central level technical working group) were always concerned about Manhiça because this is the district that Gavi would evaluate us (the country) on (Central Level Immunization program)

#### Available resources (strongly distinguishing construct)

This was a strong distinguishing factor because the high-performing district had more financial resources for project implementation compared to the intermediate- and low-performing districts. The sites, which received insufficient project funds from central MOH, could not carry out all activities that would have been ideal for higher vaccination coverage. They had to compromise and do what they could with fewer financial resources. Social mobilization activities clearly demonstrated such effects of disparity in resource availability. The high-performing site included an extra component, beyond regular methods implemented in all demonstration districts. Regular methods included television and radio spots, health worker, and community leader discussions, while in the higher resourced district, engagement of community volunteer activists, who conducted door to door educational visits, was an additional strategy. The activists received remuneration for their work; thus, a similar strategy could not be adapted in the less resourced districts where funds were scarce or unavailable. These lower performing, poorly resourced districts also experienced shortages in vehicles, fuel, and per diems for drivers, hampering health workers’ transportation of schools.

The main challenge for the HPV project was the insufficient funds which led to non-payment of the health workers and other participants’ that is teachers and community leaders per diems. (Provincial Health Directorate)

#### Access to knowledge and information (strongly distinguishing construct)

Respondents from the intermediate- and low-performing districts talked of how teachers’ lack of training affected project implementation. Teachers in the high-performing site received training to execute various activities during preparation and implementation phases of HPV vaccination. They were trained in pre-registration of eligible girls, education for vaccine-eligible girls and their parents about the upcoming HPV campaign, location of appropriate vaccination venues in schools, queuing and management of girls during vaccination, and registration of vaccination in registries and on girl’s cards. In addition teachers were trained in monitoring vaccinated girls for adverse effects following immunization. Given all the activities that were missed where teachers were not trained, this construct strongly distinguished performance.

In the second year we were better organized and were able to train teachers and they performed better in informing the girls and their parents and we performed better on the coverage (District Health Directorate)

### Process

#### Planning (non-distinguishing construct)

This construct was non-distinguishing because respondents from all demonstration districts reported a delay in the initiation of preparation activities, a factor that negatively influenced implementation. Respondents blamed the MOH central level for sending information, funds, and materials too late, when the vaccination date was fast approaching, leaving them with limited timelines to undertake district preparatory activities. The low- and intermediate-performing districts were more affected because in their case funds and materials arrived after they had commenced vaccination activities. While the construct was not distinguishing, it did influence implementation negatively.

The problem with the late arrival of funds was with central level and this didn’t improve even in the second year. The funds should arrive before, at least 3 weeks in advance in order for us to organize our teams better. (Provincial Health Directorate)

What needs to be improved is to start social mobilization early…..the information arrived late to the population because we received the social mobilization materials from the central level late (District Health Directorate)

#### Opinion leaders (strongly distinguishing construct)

This construct was strongly distinguishing and was only reported by KI respondents from the low-performing district where organizers inadvertently excluded, an important opinion leader group, mosque imams. The high- and intermediate-performing districts did not experience similar problems despite excluding religious leaders from social mobilization activities, because of the predominance of Christianity.

Religious leaders were more important because our community is largely Muslim. We heard people say that they only heard the message from the radio and not from their imams in the mosque (District Health Directorate)

#### Champions (not a distinguishing construct)

The HPV vaccine demonstration project implementation in all the sites was positively influenced by of the engagement of Mozambique’s first lady who identified cervical cancer as one of her legacy campaigns. Her involvement drove the inclusion of the two non-Gavi-funded districts in the demonstration project and facilitated the successful realization of the added demonstration projects despite limited funding. Additionally, there was high public visibility of the vaccine’s introduction due to wide media coverage of the launch ceremony that she officiated, a factor that contributed to increased demand generation in all sites.

#### Key stakeholders (strongly distinguishing construct)

Gavi’s role as donor was a strongly distinguishing construct. Gavi, alone, decided that just one demonstration site of the three proposed by Mozambique, would be funded. The unintended consequence of excluding these two districts from official Gavi-support, resulted in insufficient funds, a factor that hindered implementation success.

#### Innovation participants (strongly distinguishing construct)

Engagement of innovation participants was compromised by community beliefs in the low-performing district. According to KIs from this district, people did not understand why the vaccine was being offered to only girls instead of all children as is done with other vaccines familiar to the population.

There was a misbelief that we were vaccinating girls to make them sterile not be able to have children. It even reached a point when the girls were no longer going to school (District Health Directorate)

#### Executing (neither a distinguishing nor an influencing construct)

Despite a number of challenges, the delivery of all forecasted demonstration project HPV doses was accomplished within planned timelines in all three sites. Overall, the country’s objective of implementing a HPV vaccination pilot, in three different districts that represented three different contexts, was met.

#### Reflecting and evaluating (not a distinguishing construct)

KII participants from all sites discussed applying lessons learnings from the first-year implementation to improve performance during the second year of the project.

Comparing the first and second years of HPV vaccination, we got better in the second year because we learnt from our experience. We had created a mechanism for mobilizing the community and we had better results than in the first year (District Health Directorate)

## Discussion

Our study has revealed eight significant drivers of implementation success or failure, deduced from a thematic aggregation of findings of CFIR constructs, that either distinguished or influenced implementation performance. They are adaptability, complexity, financial resources, organizational culture and workers beliefs about the innovation, training, intervention recipients’ perceptions, engaging the right opinion leaders, and decentralization of planning processes.

The first two key drivers emerged from the two strongly distinguishing constructs *adaptability* and *complexity* which belong to the first CFIR domain of *innovation characteristics.* The delivery model, designed at MOH central level, was primarily school based and did not cater to differing levels of adolescent girl school enrollment, in disparate districts. Vaccination guidelines received by all demonstration districts, allocated a uniform 1-week period for implementing predominantly school-based vaccination, buffered by a few community visits as needed. There was no possibility for districts that had fewer proportions of school-enrolled girls to deviate from this timeline. The low-performing district had only 40% of girls enrolled in school and health workers here cited a lack of clear timelines and guidance on how to reach girls out of school as a key barrier to achieving higher vaccination coverage. Our findings are consistent with those of other published studies showing that reaching out-of-school girls is a key significant challenge to successfully implement HPV vaccination in LMICs [29] and especially in sub-Saharan Africa where an estimated 18.6 million girls of school age are unenrolled [30]. An adaptable model that could allow such districts to allocate more time and resources to community outreach visits is desirable.

The other innovation characteristic domain that impacted negatively on performance was complexity. While a complex intervention poses challenges for the health system it also offers an opportunity for the nurturing and creation of new implementation ideas. Complexities that were highlighted by health workers were those of having to administer the HPV vaccine predominantly out of health facilities and therefore having to rely on non-health workers, teachers in schools, and leaders in communities, in order to reach out-of-school girls. This brought on new types of challenges, including unclear roles and duties in the context of multiple implementing entities, with resulting divergence and conflicts where interests differed. While it may not be possible to eliminate complexity due to HPV’s novel target group (pre-teen girls) with no established health services in most countries, complexity could be minimized by clearly defining how health workers collaborate with teachers and community leaders during campaigns. Similar findings have been documented in the literature, including in a recent study from sub-Saharan Africa which highlighted the need for effective communication between the health system and community as well as development of HPV vaccination strategies to reach out-of-school girls with HPV vaccine [30].

The *inner setting* CFIR domain identified factors that influenced HPV implementation performance. Three strongly distinguishing constructs, *structural characteristics*, *available resources*, and *organizational incentives and rewards* were related to insufficient financial resources curtailing the ability of the intermediate- and low-performing districts to achieve higher vaccination coverage. Implementation performance varied based on either the availability of funding for the demonstration project or the economic development of the district. Several studies from LMICs have documented the importance of establishing a new platform to deliver HPV vaccine to adolescent girls, which in turn increases costs of its delivery and is a key barrier to expansion of its uptake [31–33]. The findings in our study underscore the need to address inequity during national scale-up. Poorer districts require more financial resources than districts with relatively wealthier populations and stronger local economies. Health workers’ values and work ethic were evaluated by the *inner setting* constructs of *culture* and *relative priority*. While they were not distinguishing, they strongly influenced implementation in all sites. Health workers’ beliefs in the importance of vaccination as a highly efficient disease prevention strategy, coupled with the resilient and resourceful organizational culture of the Mozambican health sector, helped bolster efforts to ensure that eligible populations accessed HPV vaccine. These positive drivers of organizational implementation success should be leveraged during national scale-up, however with a caveat to avoid expecting too much from health workers in a poorly resourced health system. The final significant, strongly distinguishing construct from the inner setting domain was *access to knowledge and information*. The lack of teacher training in the intermediate- and low-performing districts compromised their ability to perform expected activities during the pilot which consequently impacted negatively on vaccination coverage. Moving forward, comprehensive skill-based trainings should be planned for all health workers, teachers, and community members who will be expected to carry out any activities related to HPV vaccination.

Constructs that delve into the recipients of the intervention also stood out in our study. We excluded the domain *characteristics of individuals* from our evaluation as we were focusing on identifying drivers and barriers to health system delivery of HPV vaccine. However, the importance and relevance of intervention recipients needs manifested itself through other CFIR domains. The construct *needs and resources* from the outer setting domain and the *innovation participants* from the process domain, both strongly distinguished performance across the different demonstration project sites. Social mobilization needs to be informed by evidence-based research while still capturing and valuing recipients’ perceptions, beliefs and attitudes [34]. Additionally, the identification of the most influential opinion leaders is key to implementation success. Failure to engage Muslim religious leaders in the highly Muslim populated demonstration district undermined social mobilization. Community members informed health workers in this district that they had not taken their girls for the HPV vaccination because messages had only been relayed via radio but not by their *imams* in the mosques. In addition, the period of social mobilization prior to and throughout implementation was deemed insufficient by interviewees.

Planning was deemed important especially at the higher level of the health system. The units of analysis in our study were the frontline and also peripheral health service provision segments. Even though planning negatively influenced performance, the impact at this level was insignificant because all districts generally accomplished the demonstration project objectives despite late engagement and planning. Based on study participants’ observations, earlier involvement of districts in planning activities would likely improve implementation performance.

Our study has shown both positive and negative drivers of HPV vaccine implementation in Mozambique. These findings provide a guidance for HPV vaccination programs’ stakeholders on practices that can be replicated and those that should be avoided during scaling up of HPV vaccine delivery in the country and other LMICs with similar health and socioeconomic setting.

We found the CFIR a useful and practical tool for researching health system implementation success determinants. A salient characteristic which made it a preferable IS framework, as well as a rationale for us to recommend it to other health intervention researchers, is its unique methodology that allows for the comparison of constructs across different implementation efficiencies. This feature ensures researchers do not simply list determinants but are compelled to delve deeper and understand them in the context of implementation success or failure. Furthermore, its breadth as a comprehensive framework ensures that an extensive range of implementation determinants are considered. This usually manifests itself during the exercise of assessing all the 39 constructs and subsequently narrowing down and selecting those relevant to the evaluation. As such researchers are pushed beyond being narrow minded, irrespective of the context, their expertise, or the nature of their intervention, be it a health system or community-based intervention. Additionally, it allows for flexibility without compromise, for example, in our study we found it was possible to indirectly explore innovation recipients, despite excluding the *characteristics of individual* domain from the evaluation.

Finally, due to its widespread application, researchers are able to compare results with other studies that have applied the CFIR to implementation research evaluations. We were able to compare our findings with those of other studies that utilized the CFIR for similar type evaluations. The constructs that we selected were similar in number and scope to those of other studies [18, 19].

Our study had potential limitations. Given that interviews were being audio recorded and the political sensitivity of some of the interviews’ contents, there was a possibility of social desirability bias. We addressed this weakness by presenting participants with written information detailing how the anonymity of the data would be maintained. In addition, the interviewers were well trained and signed confidentiality agreements prior to conducting interviews. We acknowledge that the representativeness of three districts in a country of 150 districts may be limited, and care should be taken in interpreting the generalizability of our findings. Nevertheless, the relatively large number of carefully selected study participants allowed for an in-depth illustration of diverse administrative, cultural, political, and geographical differences thereby providing important learnings that are useful in informing HPV Vaccine introduction in the country and the region. Because innovation participants were not interviewed, it was not possible to triangulate findings regarding them that emerged from frontline health workers.

## Conclusion

HPV vaccination presents a pertinent opportunity for the prevention of cervical cancer in Mozambique, sub-Saharan Africa and other LMICs. However, important barriers to broad-scale implementation across heterogeneous, implementing sites, such as those highlighted in our study, need to be addressed in order for HPV vaccination to be scaled up in an effective, efficient, and expedited manner. Time lost during the preparation period for national expansion translates to a lost opportunity to curtail preventable deaths, especially given the high cervical cancer mortality rates in Mozambique and across the region. We recommend that the MOH garner local and international support to develop strategies that take into account the implementation barriers outlined in this study when planning for countrywide expansion.

## Additional file

Additional file 1: **Table S1.** CFIR construct rating rules. (DOCX 13 kb)

## Abbreviations

CFIR: Consolidated Framework for Implementation Research
HPV: Human papillomavirus
INE: Instituto Nacional de Estatística
IS: Implementation Science
KI: Key Informant
KIIs: Key Informant Interviews
LMICs: Low- and middle-income countries
MOH: Ministry of Health
NGO: Non-governmental organization
NIP: National Immunization Program
WHO: World Health Organization
