# Supplementary material for: Human papillomavirus vaccine delivery in Mozambique: identification of implementation performance drivers using the Consolidated Framework for Implementation Research (CFIR)
Source: Implement Sci. 2018 Dec 13;13:151. doi: 10.1186/s13012-018-0846-2 (PMC6293623; doi:10.1186/s13012-018-0846-2)
Supplement: Supplementary file 1 — Table S1. CFIR construct rating rules. (DOCX 13 kb) [file 13012_2018_846_MOESM1_ESM.docx]

**Table S1**

Table S1: CFIR construct rating rules

| Rating | Criteria |
| --- | --- |
| -2 | The construct is a negative influence in the organization, an impeding influence in work processes, and/or an impeding influence in implementation efforts. The majority of respondents describe explicit examples of how the key or all aspects (or the absence) of a construct manifests itself in a negative way |
| -1 | The construct is a negative influence in the organization, an impeding influence in work processes, and/or an impeding influence in implementation efforts. Respondents make general statements about the construct manifesting in a negative way but without concrete examples: (1) the construct is mentioned only in passing or at a high level without examples or evidence of actual, concrete descriptions of how that construct manifests; (2) there is a mixed effect of different aspects of the construct but with a general overall negative effect; (3) there is sufficient information to make an indirect inference about the generally negative influence; and/or (4) judged as weakly negative by the absence of the construct. |
| 0 | A construct has neutral influence if: (1) it appears to have neutral effect (purely descriptive) or is only mentioned generically without valence; (2) there is no evidence of positive or negative influence; (3) credible or reliable respondents contradict each other; and/or (4) there are positive and negative influences that balance each other out, the construct has some positive influence whereas other influences are negative and, overall, the effect is neutral. |
| +1 | The construct is a positive influence in the organization, a facilitating influence in work processes, and/or a facilitating influence in implementation efforts. Respondents make general statements about the construct manifesting in a positive way but without concrete examples: (1) the construct is mentioned only in passing or at a high level without examples or evidence of actual, concrete descriptions of how that construct manifests; (2) there is a mixed effect of different aspects of the construct but with a general overall positive effect; and/or (3) there is sufficient information to make an indirect inference about the generally positive influence. |
| +2 | The construct is a positive influence in the organization, a facilitating influence in work processes, and/or a facilitating influence in implementation efforts. The majority of respondents describe explicit examples of how the key or all aspects of a construct manifests themselves in a positive way. |
| Missing | Respondent(s) were not asked about the presence or influence of the construct or, if they were asked about a construct, their responses did not correspond to the intended construct and were instead coded to another construct. Respondent(s)’ lack of knowledge about a construct does not necessarily indicate missing data and may instead indicate the absence of the construct. |
